# Fibrotic Signaling in Cardiac Fibroblasts and Vascular Smooth Muscle Cells: The Dual Roles of Fibrosis in HFpEF and CAD

**DOI:** 10.3390/cells11101657

**Published:** 2022-05-17

**Authors:** Julian C. Bachmann, Simon J. Baumgart, Anna K. Uryga, Markus H. Bosteen, Giulia Borghetti, Michael Nyberg, Kate M. Herum

**Affiliations:** Research and Early Development, Novo Nordisk A/S, Novo Nordisk Park, 2760 Maaloev, Denmark; jcfb@novonordisk.com (J.C.B.); vsbg@novonordisk.com (S.J.B.); auyg@novonordisk.com (A.K.U.); omkb@novonordisk.com (M.H.B.); gibt@novonordisk.com (G.B.); znyb@novonordisk.com (M.N.)

**Keywords:** extracellular matrix, heart failure, atherosclerosis, fibrous cap, cardiac fibroblast, vascular smooth muscle cell, signaling, TGF-*β*, Angiotensin II

## Abstract

Patients with heart failure with preserved ejection fraction (HFpEF) and atherosclerosis-driven coronary artery disease (CAD) will have ongoing fibrotic remodeling both in the myocardium and in atherosclerotic plaques. However, the functional consequences of fibrosis differ for each location. Thus, cardiac fibrosis leads to myocardial stiffening, thereby compromising cardiac function, while fibrotic remodeling stabilizes the atherosclerotic plaque, thereby reducing the risk of plaque rupture. Although there are currently no drugs targeting cardiac fibrosis, it is a field under intense investigation, and future drugs must take these considerations into account. To explore similarities and differences of fibrotic remodeling at these two locations of the heart, we review the signaling pathways that are activated in the main extracellular matrix (ECM)-producing cells, namely human cardiac fibroblasts (CFs) and vascular smooth muscle cells (VSMCs). Although these signaling pathways are highly overlapping and context-dependent, effects on ECM remodeling mainly act through two core signaling cascades: TGF-*β* and Angiotensin II. We complete this by summarizing the knowledge gained from clinical trials targeting these two central fibrotic pathways.

## 1. Introduction

### 1.1. Dual Roles of Fibrotic Remodeling in Cardiovascular Disease

Fibrosis, the accumulation of extracellular matrix (ECM), occurs in most diseases of the heart. Nevertheless, no anti-fibrotic drugs targeting the heart exist to date. This may, in part, be due to the dual role of fibrosis, being beneficial or detrimental depending on the context. The replacement fibrosis that occurs in response to myocardial infarction (MI) can be lifesaving as the rapid production of ECM substitutes dying cardiomyocytes, thereby, to some extent, maintaining the mechanical integrity of the myocardial tissue. Although myocardial function will be compromised, and these patients are at risk of developing heart failure with reduced ejection fraction (HFrEF), fibrotic remodeling prevents the ventricle from rupturing, which would otherwise lead to sudden death [1]. Thus, fibrosis is crucial for providing mechanical strength after acute injury. In other more chronic contexts, fibrotic remodeling is detrimental to tissue function. During hypertrophic remodeling, i.e., in the pressure-overloaded heart, excessive fibrotic remodeling causes stiffening of the myocardium and severely compromises the diastolic function of the heart. This type of fibrosis is called “reactive fibrosis” and presents as interstitial fibrosis (deposited between cardiomyocytes) and perivascular (deposited around the vasculature) [2]. Reactive fibrosis is thought to be a main driver for development of heart failure with preserved ejection fraction (HFpEF).

HFpEF, which accounts for more than half of all heart failure cases and is rising progressively [3,4], is defined by reduced filling of the ventricle during diastole while the contraction in systole is preserved. Myocardial stiffening is the main cause of reduced ventricular filling. Although myocardial stiffening can be caused by the impaired relaxation of cardiomyocytes [5], there is a strong correlation between myocardial extracellular volume fraction with death and hospitalization of heart failure patients [6], indicating that cardiac fibrosis is the main driver of myocardial stiffening [7,8]. Furthermore, endothelial-independent coronary microvascular dysfunction (CMD) is prominent in HFpEF patients [9], indicating that perivascular fibrosis increases microvascular resistance in this patient sub-group. Thus, there is compelling evidence that reducing cardiac fibrosis would be beneficial for HFpEF patients.

It is important to understand the complicated and heterogenous nature of cardiovascular disease, where patients often present with multiple cardiovascular complications. Particularly HFpEF patients are a heterogenic group with one or more of the following conditions: obesity, diabetes, hypertension and advanced age. These conditions are also substantial risk factors for coronary artery disease (CAD). Indeed, a recent study by Rush et al. [9] demonstrated that 51% of the studied HFpEF patients also had obstructive epicardial CAD and that this condition was unrecognized prior to the study examination.

CAD is usually caused by atherosclerosis, the formation of a plaque inside the arterial wall. Fibrotic remodeling is crucial for the stabilization and healing of the atherosclerotic plaque [10] as the extracellular matrix is the main component of the fibrous cap that separates the highly thrombogenic necrotic core of the atherosclerotic plaque from the blood. The rupture of the plaque and the subsequent formation of thrombi obstruct the flow and perfusion to parts of the myocardium, thereby causing MI, the main cause of death worldwide [11]. Typically, prone-to-rupture plaques are characterized by the presence of a large lipid-rich necrotic core enclosed by a thin fibrous cap that is poor in collagen [12], suggesting the inhibition of fibrotic remodeling and collagen production may destabilize the atherosclerotic plaque.

**Figure 1:**
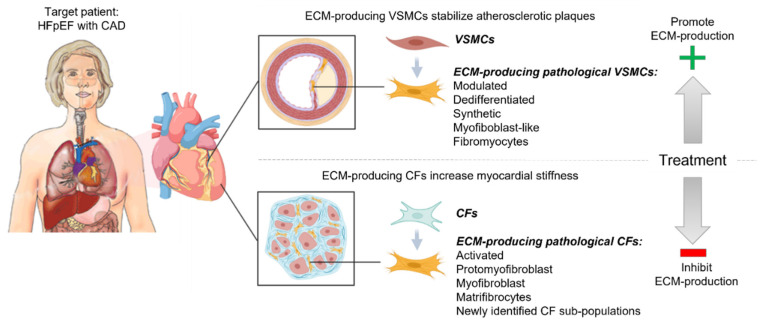
Dual roles of fibrotic remodeling in cardiovascular disease. Targeting ECM remodeling to reduce cardiac fibrosis in patients with Heart Failure with Preserved Ejection Fraction (HFpEF) and atherosclerosis-driven coronary artery disease (CAD) may entail a risk of destabilizing coronary atherosclerotic plaques, thereby causing myocardial infarction (MI). Cardiac fibroblasts (CFs) and vascular smooth muscle cells (VSMCs) regulate fibrotic remodeling of the myocardium and atherosclerotic plaque, respectively. They change phenotype in response to pathological stress and increase their production of ECM. These ECM-producing pathological CFs and VSMCs are given different names in the literature (as listed in the figure), depending on their phenotype, but may indicate similar subpopulations. These described cells are central for the induction of interstitial and perivascular fibrosis in HF patients and stabilization of the coronary atherosclerotic plaques in patients with CAD. The optimal treatment of patients with HFpEF and CAD should reduce cardiac fibrosis while maintaining, or promoting, a stable atherosclerotic plaque.

The described dual roles of fibrotic remodeling in myocardial stiffening and atherosclerotic plaque stability add another layer of complexity regarding the development of future cardiac anti-fibrotic drugs in patients with HFpEF and CAD. To clarify the similarities and differences in the regulation of the fibrotic remodeling of the myocardium and coronary atherosclerotic plaques, we review the main cell types and signaling pathways that regulate ECM production and degradation in each of these cardiac locations (Figure 1).

### 1.2. Cardiac Fibroblasts Regulate Fibrotic Remodeling of the Myocardium

Cardiac fibroblasts (CFs) are the main regulators of ECM production and maturation in the interstitial and perivascular areas of the heart. Under resting conditions, CFs maintain a healthy ECM by appropriately balancing production and degradation as well as regulating the composition and structure of the ECM [13]. Although a single unique molecular marker for CFs has not been identified, they are normally distinguished from other interstitial cells by the expression of collagen type I, the platelet-derived growth factor receptor alpha (PDGFR*α*), discoidin domain receptor tyrosine kinase 2 (DDR2) [14] and the transcription factor TCF21 [15,16,17]. In fact, TCF21 is required for the differentiation of epicardial cells into CFs [18], whereas the loss of TCF21 commits progenitors to coronary artery VSMC lineage [18,19]. Thus, TCF21 is decisive for the lineage fate of epicardial progenitor cells during development.

In response to pathological stress such as injury, chronic inflammation or changes in the mechanical environment, CFs become activated. Depending on the type of stress, fibroblast activation may involve one or more of the following functional effects: Increased proliferation, increased migration, excessive ECM production and acquisition of smooth muscle cell (SMC)-like features such as the ability to contract. Interestingly, TCF21 expression is lost in pathological conditions that induce fibrotic remodeling [20], suggesting that TCF21 may function as a master switch determining CF phenotype and pushing CFs toward an SMC-like phenotype.

The contractile property of activated fibroblasts was first described in 1971 by Gabbiani and colleagues, who introduced the cell ‘myofibroblast’ after an electron microscopy study of granulation tissue contractility. They induced contraction in strips of granulation tissue fibroblasts by agents known to contract the smooth muscle and observed functionally and morphologically characteristics close to that of SMCs [21]. Indeed, myofibroblasts are characterized by the presence of contractile *α*-smooth muscle actin (*α*SMA, encoded by the gene Acta2) filaments. However, recent data suggest that Acta2 is not required for the pro-fibrotic activity of myofibroblasts since the CF-specific deletion of Acta2 did not significantly affect contractility of myofibroblasts in vitro or cardiac repair and function following MI in vivo [22]. In agreement, early-stage myofibroblasts, named ‘proto-myofibroblasts’, are contractile despite a lack of αSMA [7]. In addition to their contractile function, myofibroblasts secrete excessive amounts of ECM proteins, such as collagens. Thus, myofibroblasts have long been known as key effector cells mediating ECM remodeling and fibrosis in most organs [7,8].

Myofibroblasts are suggested to derive from multiple cell types, including endothelial cells [23], bone marrow-derived cells and macrophages. However, compelling lineage-tracing studies using periostin (*Postn*) and *Tcf21* to label myofibroblasts and resting CFs, respectively, identified resident CFs as the main source of myofibroblasts in mouse models of heart disease [20,24,25]. Perivascular *Gli1*-positive mesenchymal stem cell-like cells are also shown to contribute to the myofibroblast pool in the mouse model of heart failure [26] and were recently confirmed in human myocardial tissue after MI [27].

Recent single-cell transcriptomics studies revealed several new CF sub-populations in the healthy and diseased hearts of animal models [28,29,30] and humans [17,31,32]. Thus, in addition to the well-studied myofibroblast, it seems that CFs are more phenotypically dynamic than previously thought. E.g., two new fibroblast sub-populations, *Cilp*-positive and *Thbs4*-positive, were identified in angiotensin II (Ang II)-infused mouse hearts where *Cilp*-positive were the most fibrogenic cell type of the two. Both *Cilp*- and *Thbs4*-positive fibroblasts populations were not identified as myofibroblasts based on the lack of *Acta2* expression [30,33], thus, questioning the traditional view that myofibroblasts are the main protagonists in cardiac fibrosis. Furthermore, a sophisticated lineage tracing study by the Molkentin lab described the dynamic differentiation path of CFs during the course of cardiac remodeling following MI [34]. Interestingly, myofibroblasts further differentiated into a new stable phenotypic state referred to as matrifibrocytes, which were also present in the mature scars of patients with MI. Matrifibrocytes were characterized by the expression of ECM genes typically expressed by chondrocytes and osteoblasts (e.g., Comp and Chad), suggesting an adaptive response to the highly stiff collagenous fibrotic environment. Along these lines, CFs were found to adopt an osteoblast cell-like fate during heart muscle calcification [35]. Thus, it is becoming increasingly clear that CFs adopt several phenotypes that change dynamically during the course of the disease.

Convincing human single-cell RNAseq (scRNAseq) of diseased human hearts, with sampling more than three patients and having a sufficient cell depth, is rare. A recent publication by Koenig et al. laid the foundation and will hopefully be followed by two major studies this year [27,36] (bioRxiv). Interestingly, RUNX1 and Gli1 are highlighted as important transcription factors (TF) for myofibroblast differentiation [27]. Together with the findings in mice, it points towards the conserved disease role of Gli1 in myofibroblast differentiation and adds RUNX1 as a driver in humans. On the contrary, TCF21 was not reported as a marker of activated CFs [27,36]; thus, TCF21 might be a unique driver in mouse myofibroblast differentiation or important for the early onset of disease. It will be interesting to see if CF sub-populations produce ECM with specific compositional profiles and whether these activities can be recapitulated in vitro. In this review, we focus on CF signaling pathways that increase ECM production in general during disease, regardless of CF phenotype and the specific type of ECM (Figure 1).

### 1.3. Vascular Smooth Muscle Cells Regulate Fibrotic Remodeling in Coronary Atherosclerotic Plaques

In the healthy state, VSMCs reside in the medial layer of the arterial wall and are defined by their contractile phenotypic state, which is essential for the regulation of vascular tone and structural integrity. They have minor proliferative and ECM-producing activity, secreting only low amounts of ECM components such as elastin, collagen and proteoglycans to provide structural support and elasticity to the vessel wall [37].

Although healthy VSMCs display a highly differentiated contractile state, they retain remarkable plasticity and, in response to pathological stimuli, undergo phenotypic switching [38,39] that is characterized by the loss of contractile markers, including *ACTA2*, SM myosin heavy chain 11 (*MYH11*) and SM22α (also referred as transgelin, TAGLN), and increase in their ability to produce ECM, proliferate and migrate [40,41].

The emergence of lineage-tracing transgenic mice enabled cell-type-specific labeling and fate mapping of VSMCs in different disease models regardless of the presence or absence of contractile markers [42,43,44]. These studies revealed that in atherosclerotic lesions, VSMCs might adopt several phenotypic states, including an osteo-chondrogenic phenotype that is present in advanced atherosclerotic plaques with ongoing calcification and a fibroblast-like phenotype, characterized by the reduced expression of contractile genes (ACTA2) and increased expression of the small leucine-rich proteoglycans (SLRPs), lumican, decorin and biglycan [19,43,45]. To illustrate the opposite phenotypic trajectory to fibroblast-derived myofibroblasts, this VSMC-derived phenotype was named a ‘fibromyocyte’. Considering the common features in CF-derived myofibroblasts and VSMC-derived fibromyocytes, it is tempting to speculate that there exists a continuous phenotypic spectrum ranging from CFs to VSMCs, where CF- and VSMC phenotypes move toward each other during disease. Indeed, the expression of the matricellular genes POSTN and SPP1, which are known to be expressed by activated CFs [46,47], was identified as key drivers of VSMC phenotypic switch in human atherosclerosis [48]. However, it is unclear to what extent VSMC-derived “fibromyocytes” exhibit a similar transcriptional profile to CF-derived myofibroblasts [19,45].

*Tcf21*, the transcription factor known to orchestrate differentiation of epicardial progenitor cells to either coronary artery VSMC or CF lineages, is central for phenotypic changes of both cell types during disease. Thus, Tcf21 induces VSMC switching into “fibromyocytes” [19,45], while the loss of Tcf21 expression is associated with the activation and differentiation of CFs into myofibroblasts [34]. Interestingly, *Tcf21* has also been causally linked to CAD, where its reduced VSMC expression increases the risk of cardiovascular diseases. In fact, *Tcf21* SMC-specific knock-out in ApoE^−/−^ mice inhibits VSMC phenotypic modulation and limits their presence in the fibrous cap, supporting a protective role of TCF21 in atherosclerosis [45].

Despite differences in cellular source, it is likely that the ECM-producing cells of the fibrotic heart and the atherosclerotic plaque engage overlapping signaling pathways that drive cellular differentiation and ECM remodeling. In the following section, we will describe the common signaling pathways for CF activation and VSMC phenotypic modulation resulting in increased ECM deposition and fibrosis in the heart and stabilization of the coronary atherosclerotic plaque.

## 2. Studying CFs and VSMCs In Vitro

Our current knowledge of fibrotic signaling pathways in CFs and VSMCs is, to a large extent, based on in vitro experiments. Most of these studies are performed on plastic tissue culture plates in standard growth or starvation media. Therefore, it is crucial to emphasize the marked phenotypic changes that occur under these conditions due to differences in the cellular, biochemical and mechanical environment compared to that of the native tissues.

**Figure 2:**
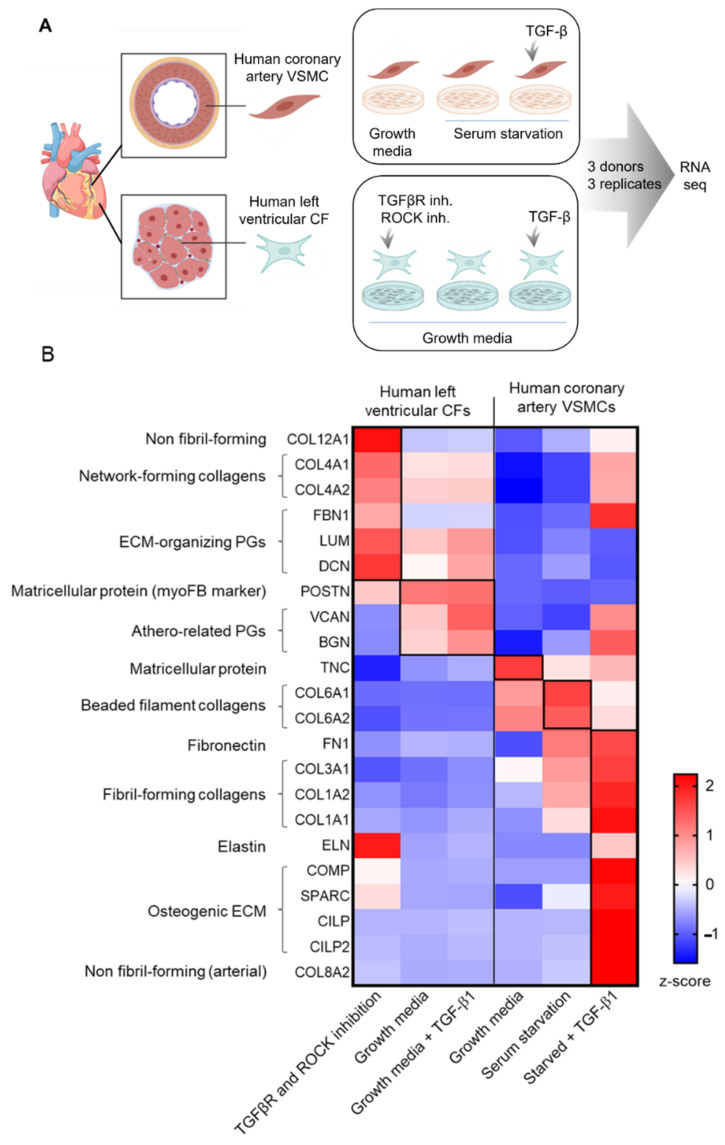
Extracellular matrix (ECM) gene expression in primary human cardiac fibroblasts and primary human coronary artery vascular smooth muscle cells varies in different in vitro phenotypes. (**A**) Schematic overview of cell types used and experimental set-up. Commercially available human primary left ventricular cardiac fibroblasts (CF, Cat# CC-2904, Lonza, Basel, Switzerland) and primary human coronary artery vascular smooth muscle cells (VSMC, Cat# 121 0612, Provitro, Berlin, Germany) from three organ donors for each cell type were seeded in 6-well plates in passage six. CFs were cultured in growth media containing 10% fetal bovine serum (FBS, Cat# 16140071, Thermo Fischer Scientific, Waltham, MA, USA) and stimulated with 10 µmol/l transforming growth factor (TGF) *β* receptor (TGF*β*R) I inhibitor (Cat# SB421542, Sigma, St. Louis, MI, USA) and 25 µmol/L Rho-associated kinase (ROCK) inhibitor (Cat# Y27632, BD Biosciences, Heidelberg, Germany) for 48 h, or 10 ng/mL TGF-*β*1 (Cat# 7754-BH, R&D Systems, Minneapolis, MN, USA) for 24 h. VSMC were cultured in growth media containing 10% FBS or in serum starvation media containing 0.5% FBS for 72 h, with subsequent stimulation with 10 ng/mL TGF-*β*1 for 24 h. For all experiments, three technical replicates were used for RNA sequencing, which was performed in parallel for all samples to minimize technical variation. RNA was isolated with Qiagen Mini Plus Kit (Cat# 74136, Qiagen, Hilden, Germany) and quality checked with a bioanalyzer (Cat# 5067-1511, Agilent RNA 6000 Nano Kit, Agilent Technologies, Santa Clara, CA, USA) before sending to Eurofins for RNA sequencing. (**B**) Heatmap of row-z scores across expression values for ECM genes after the different treatments. STAR (2.7.3) [57] was used to map samples to the GRCh38 reference genome, and mapped reads were passed to Salmon (1.2.0) [58] for transcript-level quantification on the TPM level.

CFs respond to the high stiffness of a plastic tissue dish by forming *α*SMA fibers and expressing high amounts of ECM. Since these are characteristics of activated CFs, further activation in response to profibrotic stimuli is not easy to recapitulate in vitro. Likewise, VSMCs adopt a disease-like state in vitro, losing their contractile properties and increasing the production of ECM typical for diseased arteries. Different approaches are implemented in an attempt to control in vitro phenotypes of CFs and VSMCs [49,50,51], including TGF-*β*R and ROCK inhibition to promote a resting CF phenotype [49], TGF-*β* treatment to induce the activated CF phenotype [51], serum starvation conditions to induce contractile VSMC phenotype [52,53,54] and TGF-*β* treatment to induce ECM-producing VSMC phenotype [50]. In Figure 2, we compare the ECM gene expression profiles of human ventricular CFs and human coronary artery VSMCs subjected to these treatments.

Reflecting the high basal activation state of CFs in vitro, additional treatment with TGF-*β* had only modest effects on ECM gene expression (Figure 2, original data), whereas inhibiting TGF-*β* signaling with a TGF-*β*R inhibitor and a ROCK inhibitor to promote a resting phenotype [49] had more profound effects on ECM gene expression, causing the upregulation of network-forming collagens and the small leucine-rich proteoglycans (SLRPs) decorin (*DCN*) and lumican (*LUM*). Additionally, elastin (*ELN*) and fibrillin (*FBN1*) were induced by TGF-*β* pathway inhibition in CFs (Figure 2), while there was no effect on fibril-forming collagens (*COL1A1, COL1A2* and *COL1A3*) as was previously demonstrated in freshly isolated mouse CFs [49]. This discrepancy is likely due to species differences or the longer culturing time of the human CFs presented here.

In contrast to CFs, VSMCs responded to TGF-*β* with the upregulation of *COL1A1, COL1A2* and *COL2A1* (Figure 2), important components of the fibrous cap. However, genes expressing the beaded filament collagen, collagen VI, were downregulated, demonstrating that collagen families are uniquely regulated, thus, enabling the fine-tuning of ECM structural properties. Proteoglycans such as biglycan and versican were also upregulated (Figure 2). Although these proteoglycans may contribute to stabilizing ECM [55], they also facilitate plaque destabilization by retaining positively charged lipoproteins in the intima through interaction with their negatively charged glycosaminoglycan (GAG) chains [56]. TGF-*β* also upregulated genes related to contraction (*ACTA2, MYH11, TAGLN* and *CNN1*; data not shown). This was even more prominent than the effect of serum starvation which is known to induce the contractile VSMC phenotype [52,53,54].

These original data demonstrate the importance of understanding the phenotypic characteristics of CFs and VSMCs when designing in vitro experiments and interpreting in vitro data. Due to the challenge of generating simple cell culture systems that mimic the highly dynamic and complex niche of native tissues, standard cell culture conditions are still the most common. However, if the above-mentioned limitations are considered when interpreting data, results from such in vitro studies can still provide important insight into the key signaling pathways that regulate fibrotic activity in these cell types.

## 3. Signaling Pathways That Drive ECM Remodeling in CFs and VSMCs

Pathological ECM remodeling by CFs and VSMCs is triggered by multiple cues, many of which are common for both cell types. Damage-associated molecular patterns (DAMPs) released from dying or dead cells following injury [7,59], cytokines and growth factors that are elevated in conditions of chronic inflammation such as diabetes and obesity [60,61,62] and mechanical stress caused by hemodynamic changes [63,64] are some of the main triggers for pro-fibrotic disease progression in the myocardium and vasculature. Here we review the main signaling pathways that induce ECM remodeling in CFs and VSMCs, many of which display extensive overlaps and cross-talk.

### 3.1. TGF-β Signaling

Transforming Growth Factor (TGF)-*β* is a growth factor that acts on a variety of cell types, including CFs and VSMCs, regulating inflammation, cell growth, differentiation, apoptosis and ECM synthesis. The pro-fibrotic effect of TGF-*β* has been extensively demonstrated in vivo and in vitro [51,65,66,67]. However, TGF-*β* signaling may have different effects on the pathogenesis of heart failure and atherosclerosis [67,68].

TGF-*β* signaling is initiated upon receptor binding, which facilitates the formation of a heterotetrametric complex consisting of two TGF-*β* type 1 receptors (TGF-*β*R1) and two TGF-*β* type 2 receptors (TGF-*β*R2). This results in phosphorylation of the receptor complex [69], thereby activating downstream canonical or non-canonical signaling pathways, which may lead to increased ECM production and fibrosis (Figure 2). Canonical signaling is mediated by Smad proteins, downstream effector proteins, which are subclassified into different groups depending on their functions [69]. The receptor-regulated R-Smads (Smad1, 2, 3, 5 and 8) bind and form a protein complex with common Smad (Smad4) in the cytoplasm that translocates to the nucleus and induces transcription of target genes directly or by interacting with other transcription factors. TGF-*β* signaling is further modified by the inhibitory I-Smads (Smad6 and 7) that compete with R-Smads for binding to TGF-*β* receptors and thereby block R-Smad signaling [8,70]. Thus, the functional effect of TGF-*β* signaling will highly depend on the contextual contribution of these players.

In CFs, Smad2 and Smad3 have been extensively proven to be central for ECM production and fibroblast phenotype [70,71]. Although TGF-*β* activates both Smad2 and Smad3 in vivo, CF-specific Smad2/3 knockout mice demonstrated that the observed effects on cardiac fibrosis and scar formation following pressure overload or myocardial infarction, respectively, were mainly attributed to Smad3 [70,71,72]. This is in agreement with studies in other models of fibrosis, such as renal, pulmonary and radiation-induced fibrosis [73,74,75]. In addition, in a recent Single nucleus assay for transposase-accessible chromatin using sequencing (snATAC-seq) study of human myocardial infarcted hearts, SMAD1 activity and accessibility increased along fibroblast to myofibroblast differentiation [27], suggesting that other R-Smads may be more important for cardiac fibrosis in humans.

Non-canonical TGF-*β* signaling pathways such as MAPK/p38 and RhoA/ROCK signaling cascades have also been implicated in cardiac fibrosis development and CF activation [66,69]. CF-specific deletion of p38 revealed a central role for this signaling pathway in regulating ECM production in a pre-clinical model of myocardial infarction and Ang II/phenylephrine-induced heart failure, reflected in reduced fibrosis and a higher rate of cardiac rupture in mice lacking p38 [76]. Additionally, TGF-*β* has been shown to regulate the conversion of CFs to myofibroblasts by increasing *α*SMA expression and incorporation into stress fibres [77]. It is likely that this effect is mediated via the mechanosensitive Rho-associated kinases (ROCKs) [78] since pharmacological inhibition of ROCK prevents the formation of αSMA stress fibres [49] and prevents TGF-*β*-induced tissue stiffening of tissue-engineered heart tissue [79]. ROCKs are serine/threonine kinases that induce actin polymerization by incorporating globular actin (G-actin) into filamentous actin (F-actin). Since G-actin binds and retains the transcription co-factor myocardin-related transcription factor (MRTF) in the cytoplasm, ROCK-mediated actin polymerization liberates MRTF and allows for nuclear translocation. In the nucleus, MRTF interacts with the transcription factor SRF leading to the transcription of pro-fibrotic [79,80] and contractile genes [81,82,83]. Indeed, MRTF/SRF are well-known controllers of contractile gene expression in smooth muscle cells [84].

TGF-*β* acts as a potent differentiation factor of VSMCs by increasing the expression of contractile genes such as *ACTA2*, *MYH11* and *CNN1* [54,85]. In agreement, the VSMC-specific deletion of TGF-*β*R leads to the dedifferentiation of a subset of contractile SMCs into mesenchymal stem cell (MSC)-like states with the potential to differentiate into other mesenchymal lineages such as osteoblasts, chondrocytes, adipocytes and macrophages [86]. These findings support a central role for TGF-*β* in promoting the contractile VSMC phenotype. Mechanistically, this is thought to occur by suppressing the expression of Krüppel-like factor 4 (KLF4) [86,87], a transcription repressor of contractile genes that is known to induce dedifferentiation of VSMCs [88]. Indeed, Shankman et al. demonstrated with SMC-specific conditional knockout of KLF4 an increase in the percentage of *Acta2^+^* positive cells in the fibrous cap and a reduced percentage of the macrophage-like SMC *Lgals3^+^* positive cells, thus, suggesting that KLF4 is a negative regulator of plaque stability and the contractile VSMC phenotype in vivo [89]. In contrast, KLF4 was found to increase the expression of TGF-*β*R1 in VSMCs and TGF-*β* in CFs, thereby upregulating contractile genes [90,91]. Thus, accumulating evidence indicates a central role for KLF4 in mediating TGF-*β*-induced phenotypic changes in both VSMCs and CFs, but conflicting evidence obscures the exact mechanisms [92].

In VSMCs, TGF-*β* regulates genes involved in ECM remodeling via canonical [93] and non-canonical signaling pathways [89]. The mechanism whereby TGF-*β* receptors differentially activate these signaling pathways is not clear, but it may involve interactions with other cell surface receptors. Indeed, direct physical interaction between TGF-*β*R2 and platelet-derived growth factor receptors (PDGFRs) or interleukin (IL) receptors have been demonstrated, thereby potentially shifting the balance between canonical and non-canonical signaling [94]. Along these lines, a higher ratio of TGF-*β*R1:TGF-*β*R2 was associated with increased canonical signaling by Smad3 and subsequent ECM production in hepatic stellate cells [95]. Interestingly, modulated VSMCs express more TGF-*β* and TGF-*β*R1 in stable versus unstable plaques and fewer TGF-*β*2 receptors in atherosclerotic lesions versus healthy vessel walls [96,97,98]. Moreover, VSMCs in the fibrous cap express Smad2, 3 and 4 which are essential for collagen gene expression, while VSMCs within the fibrofatty lesion do not [99]. These macrophage-rich plaques are typically unstable, supporting a role for TGF-*β*-induced Smad signaling in stabilizing the atherosclerotic plaque.

TGF-*β* is regulated both on the level of expression and activation. It is secreted in its inactive form as part of a large latent complex. When this complex is proteolytically cleaved or subjected to mechanical forces, TGF-*β* is released and can engage with its receptors [66]. Mechanical activation of TGF-*β* also occurs in vitro [51], which, in combination with the great mechano-sensitivity of CFs, results in CF activation when cells are grown in growth media on regular tissue culture plates. This is reflected in the modest change in TGF-*β* target genes following TGF-*β* treatment of human CFs in vitro (Figure 2, original data).

Interestingly, *CILP* was one of the few significantly upregulated genes in TGF-*β*-treated CFs, suggesting that the in vivo-identified *Cilp*-positive fibroblast in diseased mouse hearts [30] might be induced by TGF-*β*. *CILP* expression in VSMCs was also highly induced by TGF-*β*, suggesting there may be several similarities between TGF-*β*-induced phenotypes of CFs and VSMCs.

Together, these reports suggest that antagonizing TGF-*β* signaling in CFs may be effective in reducing myocardial stiffness but could also destabilize atherosclerotic plaques by preventing VSMC phenotypic switching towards an ECM-producing phenotype. However, more human in vivo studies are needed to final pinpoint the role of the TGF-*β*-induced phenotype in plaque stabilization and, thus, the effect of inhibiting TGF-*β* signaling in atherosclerosis.

### 3.2. Renin-Angiotensin-Aldosterone System (RAAS)

RAAS is a central regulator of the physiological and pathological responses of the cardiovascular system. The primary effector peptide Ang II [7,100,101] acts as a ligand for the two G-protein-coupled receptors (GPCRs), angiotensin type 1 receptor (AT1R) and type 2 receptor (AT2R), which are widely expressed in many tissues. The activation of the two receptors has opposing effects; signaling meditated through AT1R is involved in fibrosis, hypertension, inflammation and proliferation, whereas the AT2R acts as a negative regulator to these pathological responses [7]. Moreover, Ang II can be cleaved by angiotensin-converting enzyme 2 (ACE2) into Ang-(1-7), thereby causing cardiovascular beneficial effects via its receptor, Mas [100]. ACE2 and Mas are expressed in cardiomyocytes, CFs and vasculature. Mas activation may lead to anti-hypertrophic, anti-fibrotic, vasodilation and anti-oxidant effects, whereas ACE2 limits Ang II-signaling by its degradation of Ang II [101]. Thus, the net effect of Ang II on ECM production and fibrosis will depend on the relative contribution and distribution of its receptors and subsequent enzymatic processing of Ang II.

Ang II increases CF proliferation and migration, inhibits apoptosis and accelerates myofibroblast formation [102,103,104]. Thus, Ang II infusion is commonly used as a preclinical model of cardiac fibrosis and HF as it recapitulates several clinical manifestations of human disease [105]. However, there are also in vitro reports of reduced or no proliferation of CFs in response to Ang II [106,107]. These discrepancies could reflect indirect and context-dependent effects of Ang II. Indeed, proliferation was shown to increase as an indirect consequence of Ang II-induced secretion of ECM proteins causing the downstream synthesis of growth factors and inflammatory cytokines, leading to increased proliferation and migration [108].

**Figure 3:**
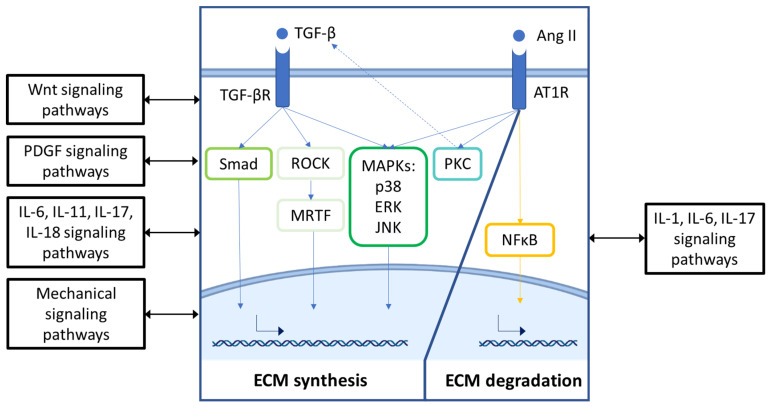
Signaling pathways that drive extracellular matrix remodeling in cardiac fibroblasts and vascular smooth muscle cells. Many signaling pathways that induce changes in gene expression, such as Wnt, Platelet-Derived Growth Factor (PDGF), interleukin (IL)-1, IL-6, IL-11, IL-17 and IL-18, and mechanical signaling, do so by affecting Transforming Growth Factor (TGF)-*β* and Angiotensin (Ang) II signaling. TGF-*β* and Ang II will, in turn, affect gene expression leading to extracellular matrix (ECM) synthesis or degradation through their multiple, and overlapping, downstream signaling pathways. Thus, TGF-*β* and Ang II are central in regulating the homeostasis of ECM turnover. NFĸB, nuclear factor kappa B; PKC, protein kinase C; MAPK, Mitogen-Activated Protein Kinase; ERK, Extracellular signal-regulated kinase 1/2; JNK, Jun N-terminal kinases; MRTF, Myocardin-Related Transcription Factor, ROCK, Rho-associated protein kinase; Smad, Suppressor of Mothers against Decapentaplegic.

The effects of Ang II/AT1R signaling on CF activation are thought to be mediated through p38 mitogen-activated protein kinase (MAPK), protein kinase C (PKC) and extracellular signal-regulated kinase (ERK) signaling cascades (Figure 3) [7,102]. These pathways directly induce fibrotic gene expression but also enhance pro-fibrotic signaling indirectly by increasing TGF-*β*1 expression [91,109,110,111]. Moreover, pro-inflammatory cytokines have also been shown to potentiate the pro-fibrotic response to Ang II by increasing AT1R expression [112], and many Ang II pathological effects have been described to occur via activation of NAD(P)H oxidases and the generation of reactive oxygen species (ROS) [100,101].

In VSMCs, Ang II acts as a multifunctional vasoactive peptide that affects both contraction and proliferation through a complex series of signaling cascades initiated by its interaction with AT1R [113]. While the vasoconstrictive effect of Ang II is short-acting and involves the mobilization of calcium from intracellular stores and subsequent contraction [114], long-acting effects on VSMCs include hypertrophy, cell proliferation, migration and increased ECM synthesis [115,116,117]. These properties are hallmarks of atherosclerotic plaque development [118] and are induced via pathways involving tyrosine receptor phosphorylation (i.e., MAPK) and non-receptor tyrosine kinase activity (i.e., Src family kinases) [113,119,120]. Ang II has also been found to induce inflammation in VSMCs by activating Nuclear Factor Kappa B (NFκB) [121,122,123], thereby upregulating the NFκB target gene, IL-6. This effect was completely abolished by AT1R blockade with losartan [124], substantiating the pro-inflammatory effects of Ang II. In VSMCs, Ang II-induced NF*κ*B also upregulates the matrix metalloproteinase (MMP)-9 which is involved in ECM degradation and plaque destabilization [125]. Thus, Ang II promotes a switch from stable plaques to vulnerable plaques in vivo, marked by a thinner fibrous cap, reduced *α*SMA positive cells and increased macrophage infiltration [126].

Together these studies suggest that it would be beneficial for cardiac fibrosis and atherosclerosis to inhibit Ang II signaling in CFs and VSMCs. However, as we will discuss in Section 3.3., despite the reduction in cardiac fibrosis, RAAS blockade therapies have failed to demonstrate beneficial effects in patients with HFpEF, suggesting that blocking these pathways alone is insufficient to preserve cardiac function in these patients [127].

### 3.3. Wnt/β-Catenin Signaling

Wingless-INT (Wnt) signaling is also implicated in both cardiac fibrosis and atherosclerosis by mechanisms relating to myofibroblast-like cell differentiation [66,128,129].

Wnt ligands are secreted glycoproteins that act on a family of transmembrane receptors called Frizzled (Fz). Upon ligand binding, Fz receptors form a receptor complex together with the co-receptors low-density-lipoprotein-related receptor-5 and -6 (LRP5 and LRP6) at the plasma membrane. Once activated, the Fz/LRP complex can trigger both canonical and non-canonical signaling cascades [128]. The canonical Wnt/*β*-catenin signaling pathway activates the Disheveled (DVL) protein which inhibits the “destruction complex” that normally degrades *β*-catenin, resulting in the stabilization, accumulation and nuclear translocation of *β*-catenin, where it activates target genes [130,131,132]. Wnt/non-canonical signaling includes pathways involving MAPK, Rho GTPases or calcium release from intracellular stores [133], which subsequently affects gene expression by activating calcium-activated transcription factors [134,135].

Negative regulation of Wnt signaling can occur at several levels, such as direct inhibition by Wnt Inhibitory Factor-1 (WIF1) or Secreted Fz-related proteins (SFRPs) that prevent Wnt interaction with receptor complexes [136]. Among other important inhibitors are the Dickkopf (Dkk) proteins (Dkk1, Dkk2 and Dkk4) and Sclerostin, which all antagonize Wnt signaling by binding to the LRP receptors and thus blocking binding sites. Dkk1, Dkk2 and Dkk4 all antagonize Wnt canonical pathways, while the role of Dkk3 is not clear but mainly activates the canonical pathways [137,138].

Fibrotic tissues have higher levels of Wnt proteins along with decreased levels of the Wnt signaling antagonist Dkk1 compared to healthy tissues, suggesting a role in fibrotic remodeling. This expressional regulation seems to be mediated by TGF-*β,* which was found to downregulate Dkk1 in a p38-dependent manner [139]. In agreement, TGF-*β*-induced Wnt secretion caused fibroblast activation and fibrosis development [140], suggesting that pro-fibrotic effects of Wnt are regulated by TGF-*β*. On the other hand, Wnt signaling can also promote fibrosis by inducing TGF-*β* signaling. The Wnt ligand, Wnt3a, was shown to up-regulate TGF-*β* and Smad2 in a *β*-catenin-dependent manner [141] and increase *Tagln* and *Acta2* in convergence with TGF-*β*1/Smad signaling, thereby promoting myofibroblast differentiation [142].

Although the role of Dkk3 on Wnt signaling remains unclear, it has been shown to activate the canonical and inhibit the non-canonical Wnt pathways in the heart, exerting beneficial effects on heart function, and cardiac fibrosis as shown in studies using Dkk3-genetically modified mice subjected to pressure overload [143], Ang II infusion [144] or MI [145]. These cardioprotective effects of Dkk3 are exerted via the inhibition of the Wnt non-canonical ASK1/JNK/p38 signaling pathways [143,145] and has led to the proposal of Dkk3 as a therapeutic target for the treatment of heart failure [146].

Arterial expression of the Wnt signaling antagonist Dkk1 is increased in atherosclerotic lesions compared to healthy arterial segments [147] and has been proposed to promote atherosclerotic plaque formation and instability [148]. However, results from mouse models of atherosclerosis were discouraging, showing no effect of Dkk1 inhibition with neutralizing antibodies on plaque lesion area [147]; alas, lesion size is not a suitable surrogate for plaque stability [149]. This may, in part, be due to the limited translational value of these models as they do not fully recapitulate human atherosclerotic disease and plaque rupture is rarely observed [150]. Indeed, in contrast to patients, Dkk1 expression was almost absent in atheroprone arterial segments in these murine models of atherosclerosis [147].

Despite the possible protective role of Dkk3 in the heart, studies of atherosclerotic development have shown contradicting results [151,152,153]. One study showed a reduced number of VSMCs, collagen and elastin in the fibrous cap of Dkk3^−/−^ ApoE^−/−^ mice, leading to a vulnerable plaque phenotype. In agreement, Dkk3 induced the differentiation of vascular progenitor cells into VSMCs and potentiated TGF-*β*-induced ECM in vitro [153]. In contrast, another study also using Dkk3^−/−^ ApoE^−/−^ mice showed an increased number of VSMCs and increased collagen content in plaques [152]. Thus, more mechanistic studies are needed to fully elucidate the effects of Dkk3 in plaque development and stability. Moreover, Dkk3 has shown detrimental effects in kidney fibrosis [154,155], further indicating distinct roles of Dkk3 in the pathogenesis of different organs.

Together, these findings suggest that the Wnt signaling pathway, depending on its mediators, may be involved in the differentiation of both CFs and vascular progenitor cells into a myofibroblast-like ECM-producing phenotype, thereby affecting atherosclerotic plaque stability and cardiac fibrosis progression. However, the pathway is complex, and the contradictory results mentioned above highlight a need for caution when targeting specific pathologies.

### 3.4. PDGF Signaling

PDGFs are involved in several pathogeneses, including cardiac fibrosis and atherosclerosis. They stimulate proliferation and direct migration, differentiation and physiological functions in several mesenchymal cell types, such as fibroblasts, VSMCs and pericytes [156,157].

The PDGFs (-AA, -BB, -AB, -CC and DD) are homo- or heterodimeric growth factors that signal by binding to and activating two different receptor tyrosine kinases PDGFR*α* and PDGFR*β* [158]. In the adult mouse heart, PDGFR*α* is expressed by fibroblasts and PDGFR*β* by pericytes and smooth muscle cells. The binding of PDGFs induces receptor dimerization and transphosphorylation, triggering several downstream signaling cascades, including phospholipase C *γ* (PLC*γ*), PI3K, MAPKs and Signaling Transducers and Activators of Transcription (STATs), leading to the regulation of target genes involved in cell proliferation, differentiation, survival and motility [157]. PDGFR*β* is mainly implicated in blood vessel formation and kidney development, and the disruption of either PDGFR*β* or PDGF-BB genes leads to premature death in mice due to the lack of several cell types such as smooth muscle cells, pericytes and mesangial cells [159,160,161]. In the heart, PDGFRα is uniquely expressed in adult CFs and in vitro inhibition of PDGFRα results in fibroblast apoptosis, whereas stimulation promotes cell survival [162]. The PDGF ligands bind to multiple PDGFRs, except PDGF-DD, which only binds PDGFRβ [163]. PDGF-DD deficient mice are viable with only mild circulatory defects and a modest increase in blood pressure [164], indicating some degree of redundancy among PDGF ligands.

PDGF-AA/PDGFR*α* signaling in in vitro cultures of CF increased collagen type I and III along with *α*SMA, suggesting myofibroblast differentiation, a response which was inhibited by the PDGF inhibitor imatinib [165,166]. Moreover, transgenic mice overexpressing either PDGF-DD or PDGF-CC develop cardiac fibrosis marked by fibroblast proliferation [156,167]. These pro-fibrotic actions are largely mediated via the TGF-*β*1 signaling pathway [166] but may also be influenced by crosstalk with Ang II signaling pathways since PDGFRs are known to interact directly with the AT1 receptor [100] (Figure 3).

PDGFs also induce phenotypic switching of VSMCs from a contractile to a synthetic/proliferative phenotype [156], supporting a central role during atherosclerotic plaque development. This effect is achieved by the downstream repression of myocardin/SRF, the main transcription factor of contractile genes. PDGFR*β* signaling causes the phosphorylation of ELK-1, which then competes with myocardin for the same docking site on SRF, thereby inhibiting myocardin/SRF interaction and function [168]. Furthermore, PDGF-BB increases the expression of KLF4, which inhibits the myocardin/SRF-induced transcription of contractile genes [88,169].

While inhibiting PDGF signaling pathways involved in fibrosis would likely be beneficial in the context of cardiac reactive fibrosis, it is less clear how this would affect the stability of the atherosclerotic plaqueA recent GWAS study identified *PDGFRA*, the gene encoding PDGFR*α*, as a CAD-associated gene, with high expression of *PDGFRA* in coronary arteries being associated with a higher risk of CAD [170]. Thus, human genetic data suggest beneficial effects of inhibiting PDGF signaling in atherosclerotic patients. Although phenotypic switching of contractile VSMCs to proliferative/synthetic VSMCs causes intimal thickening and plaque progression, it may also be temporarily beneficial for plaque healing and fibrous cap development [171,172]. Thus, the final effect of inhibiting PDGF signaling will likely depend on the time of intervention.

### 3.5. Interleukins

Interleukins (IL) are a family of important pro-inflammatory and anti-inflammatory cytokines that are expressed in a wide range of tissues by a variety of cells, including those of the heart and vasculature [173]. Both VSMCs and CFs have multiple IL-receptors which regulate their state and functions [174,175]. Recent studies have shown the therapeutic potential of inhibiting signaling by certain ILs in CFs and VSMCs [176,177] as it may relieve inflammation and fibrosis. We here focus on five interleukins (IL-1, IL-6, IL-11, IL-17 and IL-18) that are of particular interest with regard to the regulation of ECM remodeling by CFs and VSMCs of the heart.

#### 3.5.1. IL-1

IL-1 has two distinct isoforms, IL-1*α* and IL-1*β*. IL-1*α* is an intracellular cytokine that is only released when cells are damaged or undergo necrosis [178], i.e., dead cardiomyocytes following injury [179,180] and in the necrotic core of plaques [181]. Unlike IL-1α, which is secreted in its active form, IL-1*β* is activated by the inflammasome and secreted from cells in response to specific pathological stimuli [182]. Receptor binding of either ligand leads to activation of downstream signaling pathways, including the MAPK (ERK, p38 and JNK) and NF*κ*B cascades, ultimately leading to a pro-inflammatory response [178]. CFs are highly responsive to IL-1, which induces an ECM-degrading program and delays myofibroblast differentiation [178,183,184]. Interestingly, fibroblast-specific deletion of the IL-1R, but not IL-1*α*, resulted in reduced cardiac remodeling after MI [185], suggesting that IL-1*α* might not be an important regulator of cardiac remodeling, but likely other IL-1s interacting with the IL-1R.

Not so surprisingly, a similar response to IL-1s is observed in VSMCs. IL-1 stimulation in VSMCs has demonstrated increased proliferation, migration and importantly, ECM-degradation (via upregulation of MMP-2, -3 and -9) together with a sustained inflammatory response [175,186,187]. Both apoptosis and necrosis occur in advanced plaques versus normal vessels, and levels are increased in unstable versus stable plaques. Necrotic VSMCs release both IL-1*α* and IL-1*β,* which direct further inflammation (via IL-6 and MCP-1) and lead to an unstable plaque phenotype [188]. The evil wheel keeps spinning; myocardial infarction occurs when coronary plaques rupture, causing further inflammation in the heart. Thus, targeting inflammation may serve as a promising target in reducing fibrosis and stabilizing plaques.

In the Canakinumab Anti-inflammatory Thrombosis Outcomes Study (CANTOS) (NCT01327846), the specific inhibition of IL-1β in patients with previous AMI and evidence of systematic inflammation resulted in a reduction of major cardiovascular events, independent of lipid-lowering [189]. Among the 10 061 patients enrolled in this trial, 385 had a heart failure hospitalization event (HHF) during the follow-up period. Data collected on this patient population suggests that the treatment is related to a dose-dependent reduction of HHF and HF-related mortality [190].

Given the pleiotropic and immunomodulatory effect of IL-1*β*, it is difficult to establish if the beneficial effects observed after systemic inhibition could be partly due to a direct effect on CFs and VSMCs.

#### 3.5.2. IL-6

IL-6, another important cytokine which recently emerged as a pivotal factor for the development of HFpEF [191] and atherothrombosis [192], shows promising therapeutic target potential. IL-6 is produced by a variety of cells, including cardiomyocytes, CFs and VSMCs [193]. Many factors induce IL-6 secretion in CFs and VSMCs, such as cytokines, growth factors and biomechanical stress [194,195,196,197,198]. IL-6 induces myofibroblast differentiation of CFs, leading to cardiac fibrosis and promoting hypertrophy in cardiomyocytes [199,200]. In addition, both hypoxic conditions and macrophages induce IL-6 secretion, which further drives TGF-*β*/Smad3 signaling in CFs, leading to myofibroblast formation and upregulation of MMPs, such as MMP-2 and MMP-9 [201,202]. However, IL-6 has different downstream signaling pathways [203], likely contributing to the contradictory results seen in vivo. Indeed, the activation of the main downstream mediator, STAT3, has been suggested to play a key role in cardio-protection [203,204]. While excessive IL-6-dependent signaling promoted cardiac inflammation and adverse remodeling [205], the complete lack of IL-6 did not rescue cardiac remodeling or survival [206] in MI animal models. The animal models used are also likely to influence the results and pathways involved, thus making it difficult to assess the role of IL-6.

In VSMCs, IL-6 induces cell proliferation and migration [207,208], increases AT1R mRNA and protein levels [209] and reduces contractility and SM22*α* protein [195,210]. Blocking IL-6 inhibits PDGF-induced proliferation and migration of VSMCs, suggesting that IL-6 may act downstream of PDGF signaling. IL-6 signaling is complex and may show different effects depending on the timing and type of arterial disease [211]. Nevertheless, most in vivo experiments imply an athero-protective role of IL-6 inhibition [212,213,214,215,216]. This is supported by Mendelian randomization studies, which implicated the IL-6 signaling system in both atherogenesis and acute plaque rupture. Indeed, in addition to its pro-fibrotic effects, IL-6 also affects ECM degradation, which could, in turn, destabilize the atherosclerotic plaque.

Recently, the safety and efficacy of IL-6 inhibition have been assessed among individuals at high risk of atherosclerosis and kidney disease during the phase 2 RESCUE trial (NCT03926117). IL-6 inhibition by ziltivekimab, a monoclonal antibody directed against the IL-6 ligand, resulted in a dose-dependent decrease of high-sensitivity C-reactive protein (hsCRP) and other inflammatory biomarkers [192]. These encouraging data led to the long-term end-point trial ZEUS (Ziltivekimab Cardiovascular Outcomes Study, NCT05021835), the results of which are expected in 2025 [217].

#### 3.5.3. IL-11

IL-11 is a member of IL-6-like cytokines [218] and has recently gained more attention in both cardiac and liver fibrosis but also in vascular remodeling. Downstream effectors of TGF-*β*1 are proposed as promising targets for anti-fibrotic treatments with the upside of dodging upstream toxicities. IL-11 is upregulated in response to TGF-*β*1 and is shown to be required for its pro-fibrotic effects [177,219]. Both IL-11 and its receptor (IL11RA) are specifically upregulated in CFs, driving ERK-dependent signaling and resulting in fibrogenic protein synthesis. IL-11 transgene expression led to heart and kidney fibrosis, whereas IL-11RA1 genetic knockout showed protective effects against disease progression [177]. In line with these results, IL-11 neutralizing antibodies effectively reduced profibrotic responses by TGF-*β* and Wnt3a [219], suggesting its downstream role of TGF-*β* and canonical Wnt signaling. In other indications, IL-11 also induces fibrosis and inflammation via ERK-dependent pathways, such as in liver fibrosis [220] and idiopathic pulmonary fibrosis [221], thereby confirming its actions in the pathogenesis of multiple diseases.

IL-11 was also shown to be important for VSMC dedifferentiation [222]. Both IL-1*α* and TGF-*β* stimulation dose-dependently increase IL-11 production in VSMCs [223]. In a recent study, IL-11 stimulation of VSMCs resulted in a reduction of the contractile SM22*α* and MYOCD, an increase in proliferation and migration and lastly an increased secretion of TIMP1, MMP2 and collagen. These effects were also mediated via ERK-dependent signaling and indicated that IL-11 acts downstream of TGF-*β*1 and Ang II signaling [222]. In contrast, a previous in vitro study showed IL-11 to attenuate VSMC proliferation and NFκB activity [224]. However, the studies investigating IL-11 effects on VSMCs are scarce, and the results should be interpreted with caution. IL-11 may act through the same signaling pathways (mainly ERK) in modulating CFs and VSMCs towards a similar phenotype characterized by increased proliferation and ECM remodeling abilities.

#### 3.5.4. IL-17

IL-17 is secreted by various immune cells, such as TH17 cells, mast cells and neutrophils and is involved in the development of atherosclerosis and cardiac fibrosis [176,225,226,227]. The IL-17 family consists of many ligands (IL-17A-F) and receptors (i.e., IL-17RA-E) [228]. Among the most studied is IL-17A (often referred to as IL-17), which is known for its involvement in inflammatory processes [229]. Both in vivo and in vitro studies have demonstrated a role for IL-17A in promoting ECM production and fibroblast-to-myofibroblast differentiation along with an increased inflammatory profile [176,230] likely mediated via PKC/ERK1/2/NF*κ*B pathways [227]. Previous studies have also demonstrated that IL-17A enhanced the proliferation and migration of CFs and potentiated myofibroblast differentiation by promoting IL-6 secretion [231,232]. Taken together, these studies indicate IL-17A as a promising target for inhibiting the fibroblast-to-myofibroblast transition and thus the progression of cardiac fibrosis.

IL-17 serum levels are increased in patients and mouse models of atherosclerotic disease [233,234,235]. However, in contrast to cardiac fibrosis, the effects of IL-17A on plaque stability are more complex. Supporting a role for IL-17A in ECM production, the inhibition of IL-17A in atherosclerosis with neutralizing antibodies has been shown to prevent fibrous cap formation by reducing collagen accumulation [236]. However, IL-17A did not increase ECM production in a recent study by Orejudo et al. [237]. Other described effects of IL-17A include increased apoptosis [234], inflammation [238] and migration via MMP-9-dependent mechanisms resulting from NFκB and AP-1 activation [239], suggesting highly contextual roles of IL-17A.

#### 3.5.5. IL-18

IL-18 has been implicated in heart failure, and its activation of the inflammasome leads to CF activation [240]. CFs exposed to IL-18 increase migration, proliferation and ECM components such as collagen I and III and MMP-2, together with increased gel contraction likely via JNK and PI3K pathways [241]. In addition, pro-fibrotic cytokine release from activated macrophages enhances IL-18 expression in CFs, promoting phosphorylation of TGF-*β*/SMAD2/3 known to induce fibroblast-to-myofibroblast activation [242].

Higher levels of IL-18 were found in human plaques versus healthy tissues [243] and in unstable versus stable plaques [244]. In line with these findings, IL-18 overexpression in ApoE^−/−^ mice leads to vulnerable plaques by decreased collagen content and cap thickness [245,246]. In contrast to the effects on ECM production seen in CFs, IL-18 is thought to induce IFN-y production in T cells and macrophages, leading to collagen and elastin inhibition in VSMCs, thus resulting indirectly in the destabilization of plaques [247]. IL-18 receptors are elevated in VSMCs in atherosclerotic plaques, and many signaling pathways are activated by IL-18, such as Src kinase, PKC, p38 and JNK MAPKs, Akt kinase leading to activation of NFκB and AP-1 [248]. IL-18 also promotes the proliferation and migration of VSMCs [249,250], as seen in CFs. Interestingly, Ang II increases IL-18 receptor expression [248], IL-18 and MMP-9 expression together with increased migration and proliferation via AT1R, an effect that was abolished with IL-18 neutralizing antibodies [251]. In addition, VSMCs exposed to IL-18 showed decreased contractile markers ACTA2, SM22*α* and increased calcium deposition, alkaline phosphatase activity and expression of RUNX2, BMP2 and Osteocalcin, suggesting dedifferentiation towards an osteogenic phenotype [252,253]. As for many of the previously discussed cytokines, IL-18 seems to potentiate plaque vulnerability mainly via its inhibitory effects on ECM production restricted to VSMCs. In contrast, IL-18 on CFs seems to increase ECM production and induce contractile features leading to myofibroblast formation, suggesting that signaling pathways in CFs and VSMCs may lead to different fates.

### 3.6. Mechanical Signaling

Under normal conditions, the beating heart and its pulsatile arteries are continuously exposed to mechanical forces that cause the tissue to stretch and compress. Changes in these mechanical cues may result in immediate activation of the embedded cells through a process called mechanotransduction, the conversion of a mechanical stimulus into a chemical action [254,255,256]. Mechanotransduction can occur via transmembrane proteins, including integrins and syndecans (e.g., syndecan-4 [257]) and mechanosensitive ion channels (e.g., Piezo1 [258]), via cytoskeletal dynamics (e.g., ROCK [259,260]) and the liberation of ECM-stored signaling molecules in response to mechanical stress (e.g., TGF-*β* [261]). Although mechanotransduction occurs in both CFs (reviewed in [63,254]) and VSMCs (reviewed in [50,262]) and is crucial for regulating cell phenotype, the two cell types differ greatly in their baseline electrical excitability, cell–cell contacts, structural properties and mechanical environment. Thus, a direct comparison of mechanotransduction signaling pathways and cellular responses in these two cell types is challenging.

In general, CFs will respond to mechanical stress by becoming more contractile (myofibroblast differentiation) and increasing the production of ECM components. However, which ECM genes are activated will depend on the specific combination of mechanical stimuli (stiffness and stretch) as well as the initial phenotype of the CFs [62]. Thus, resting and activated CFs may respond differently to mechanical stress. In addition, the mechanical environment may affect responses to biochemical stimuli. In a recent study, CFs exposed to biochemical signals and inflammatory agents alone tended to promote protease secretion and thus matrix degradation, whereas, in combination with stretch, they shifted towards matrix accumulation [263].

When VSMCs are introduced to physiological stretch (<10% cyclic stretch) in vitro, they reduce proliferation, migration and inflammation and dedifferentiate into a more quiescent phenotype characterized by increased contractile markers such as *CNN1*, *ACTA2*, *MYH11* and *TAGLN* [50], possibly through mechanical activation of TGF-*β*1 [264]. However, when supra-physiological stretch (>10% cyclic stretch) is applied to VSMCs, they gain a more synthetic phenotype defined by increased proliferation and loss of contractile markers [50], resembling the phenotypic modulation observed during disease in vivo [40,41].

Taken together, changes in mechanical stress in the heart and vasculature induce increased ECM production in both CFs and VSMCs. This may initially be a compensatory response to resist the increased mechanical burden of the tissue. ECM remodeling will itself change the cellular mechanical environment (e.g., increase stiffness); thus, mechano-signaling and responses will be dynamic during disease development.

## 4. TGF-*β* and Angiotensin II Signaling Pathways—Center of Stage for ECM Remodeling in Both CFs and VSMCs

Despite the complexity of the described signaling pathways, the direct effect on ECM remodeling by CFs and VSMCs seems to be mediated mainly by TGF-*β* and Ang II pathways (Figure 3). Hence, although Wnt, PDGF, IL, and mechanical stimuli affect multiple functions and phenotypic characteristics of both cell types, their effects on ECM turnover are mainly mediated through these two pathways. Moreover, these pathways are highly overlapping and exert extensive crosstalk, thereby enabling context-dependent responses. Interestingly, VSMCs seem to engage the NF*κ*B signaling pathway to a greater extent than CFs, which may reflect the higher inflammatory environment of the atherosclerotic plaque.

## 5. Clinical Studies of TGF-*β* or RAAS Inhibition: Effects on Fibrotic Remodeling

Targeting fibrosis, in general, remains a major challenge due to dynamic and diverse biology [265]. Currently, idiopathic pulmonary fibrosis (IPF) remains one of the few chronic diseases with approved anti-fibrotic drugs. As depicted in Figure 3, TGF-*β* and RAAS signaling are the main pathways that induce ECM expression. Thus, inhibition of these pathways would be expected to reduce myocardial stiffness in HFpEF patients but may increase the risk of coronary plaque rupture in patients with CAD. To better understand whether the pre-clinical results reviewed above translate into patients, we summarize the learnings from clinical studies of TGF-*β* and RAAS inhibition.

### 5.1. Anti-TGF-β Treatment

In addition to the described pro-fibrotic effects of TGF-*β* in CFs, TGF-*β* signaling also affects cardiomyocyte, endothelial and immune cell functions. Not surprisingly, this pathway is implicated in the pathogenesis of cardiac disease across different etiologies. TGF-*β* is a pleiotropic factor, promoting healing in the first phase after the injury by activating fibroblasts and repressing macrophage-driven inflammation [67]. However, prolonged activation leads to excess fibrosis and promotes maladaptive remodeling. Pre-clinical evidence found TGF-*β* to be among the major drivers of the pathogenesis of MI, HF, cardiomyopathies and arrhythmia [266,267]. Increased TGF-*β* expression or activity was confirmed in human cardiac tissue harvested from patients with end-stage HFrEF [266,267], hypertrophic cardiomyopathy (HCM), and in serum from patients with hypertensive heart disease [268] and HCM [269]. Although few studies have addressed cardiac TGF-*β* expression in well-characterized HFpEF patients, it correlates with diastolic dysfunction in transplanted hearts [60]. Given the beneficial effect of TGF-*β* signaling inhibition in pre-clinical models of pressure overload [270,271,272] and the pivotal role of fibrosis in HFpEF pathogenesis [60], it is plausible that TGF-*β* inhibition would be beneficial for this patient population with a high unmet need.

As mentioned in the introduction, HFpEF syndrome and CAD co-exist and have overlapping patient segments. Thus, for safety reasons, it is crucial to examine the effect of TGF-*β* inhibition on atherosclerotic plaque stability. The contribution of TGF-*β* to the pathogenesis of atherosclerosis is quite complex and depends on the spatial and temporal context [68]. TGF-*β* is an immunomodulator whose pro- or anti-inflammatory activity depends on cell type and the inflammatory milieu [67]. Several pre-clinical studies have shown that TGF-*β* inhibition induces a pro-inflammatory, lipid-rich, collagen-poor phenotype that is characteristic of unstable plaques in humans [98]. In agreement, TGF-*β* protein levels are higher in stable compared to unstable human plaque lesions, as demonstrated by immunohistochemistry [273]. Along these lines, preoperative plasma levels of TGF-*β* are lower in patients with unstable carotid plaques than in their stable counterparts [273]. However, given the lack of reliable animal models that recapitulate plaque rupture [67], and the lack of clinical studies directly investigating the effect of TGF-*β* inhibition in a population with high risk for CAD, it is difficult to draw conclusions regarding the clinical importance of TGF-*β* for stabilizing atherosclerotic plaques.

The development of anti-TGF-*β* treatments has been hindered by serious adverse effects consequent to the treatment, probably due to the multifunctional role of this growth factor in tissue homeostasis. Fresolinumab, an antibody that neutralizes all TGF-*β* isoforms, was developed for cancer therapy and was also used in clinical studies targeting fibrotic diseases, such as scleroderma and glomerulosclerosis, with minimal benefit and development of cutaneous lesions in some patients [274,275,276]. Recently, another pan neutralizing TGF-*β* antibody has shown cardiotoxicity in non-clinical toxicology studies conducted in non-human primates [277]. Thus, long-term systemic inhibition of this pleiotropic signaling pathway may cause a broad spectrum of adverse effects. However, in order to prevent fibrosis development in chronic HF patients, early intervention and long-term treatments are likely necessary [67]. A tissue-targeting approach aimed to localize TGF-*β* inhibition specifically to cardiac tissue, together with a sound patient segmentation strategy aimed to identify patients that would benefit the most, may improve both the safety and efficacy of TGF-*β* targeted treatment.

### 5.2. Pirfenidone

Pirfenidone is a small molecule with anti-fibrotic, anti-inflammatory and antioxidant properties [278]. It is usually listed among TGF-*β* inhibitors since pirfenidone reduces TGF-*β* expression and blunts TGF-*β* signaling [279]. However, the exact mechanism underlying these actions is unknown. It was reported that pirfenidone inhibits other pro-fibrotic growth factors, such as PDGF and bFGF [279]. Pirfenidone also decreases the level of TNFα and other pro-inflammatory cytokines, such as IL-1*β*, in vivo [280,281] and in vitro [282] by an unknown mechanism. Thus, its action is not limited to directly targeting fibrosis. Indeed, it has been described in different animal models that pirfenidone limits inflammation by inhibiting macrophage infiltration [283] and T cell activation [284].

Pirfenidone is clinically approved for IPF, where it reduces disease progression and mortality [285,286]. In phase 3 clinical trials ASCENDENT [285] (NCT01366209) and CAPACITY [287] (NCT00287716 and NCT00287729), no differences were observed in cardiovascular death or non-fatal MI between patients in the pirfenidone and placebo group, and there were no specific warnings or precautions for the use of pirfenidone in patients with cardiovascular comorbidities. It is worth mentioning that this post hoc analysis was limited to a population enrolled in clinical trials where patients with unstable cardiovascular conditions in the prior six months were excluded [288]. Therefore, these results must be interpreted cautiously.

The profile of patients with IPF (>60 years of age, mostly male and frequently current or former smokers) is comparable to the most common profile of patients at higher risk for cardiovascular disease [288]. Thus, even though these clinical trials were not conducted specifically on CAD patients, and plaque burden was not measured experimentally, it is tempting to speculate that some of these patients would present with CAD and, therefore, anti-fibrotic treatment could potentially increase the risk for plaque rupture.

Pirfenidone was also recently tested as a treatment of HFpEF in a double-blinded, phase 2 trial called PIROUETTE (The Efficacy and Safety of Pirfenidone in Patients with Heart Failure and Preserved Left Ventricular Ejection Fraction, NCT02932566). Here, patient selection was not purely based on EF and natriuretic peptides levels, in contrast to previous clinical trials, but also based on cardiac MRI, thereby enabling the selection of patients specifically presenting with myocardial fibrosis. Treatment with pirfenidone resulted in significantly reduced myocardial extracellular volume with 1.21% in comparison with the placebo, but there were no differences in left ventricular diastolic function, atrial size and function or right ventricular size and function [289]. This moderate cardioprotective effect could potentially be explained by the late initiation time of intervention or redundancy of the pro-fibrotic signals. Nevertheless, this clinical trial is a great example in terms of patient segmentation and a forerunner for the ones to come [290].

### 5.3. RAAS Inhibitors

The RAAS system is a primary regulator of body homeostasis, regulating blood pressure, fluid and electrolyte balance [291]. Maladaptive regulation of this system is deeply involved in the pathogenesis of cardiovascular disease. RAAS inhibitors are considered a cornerstone in cardiovascular management. Drugs falling in this category are ACE inhibitors (ACE-I), Ang II receptor blockers (ARB) inhibiting AT1R, renin-inhibitors, mineralocorticoid receptor antagonists (MRA) and the “new kid on the block”, the combination of ARB/neprilysin (NEP) inhibitors (ARNi) [292].

In hypertensive patients, both ACE-I and ARB treatment reduces myocardial fibrosis measured in endomyocardial biopsies [293,294]. ARB treatments have been correlated with the attenuation of the progression of fibrosis in hypertrophic cardiomyopathy [295] (NCT01150461) and atrial fibrillation [296]. MRAs also show anti-fibrotic effects in studies of patients with metabolic syndrome [297] and left ventricular diastolic dysfunction [298]. However, the use of this drug is associated with the risk of hyperkalaemia [299]. It is worth mentioning that, although these clinical studies demonstrate that RAAS blockade reduces cardiac fibrosis, the number of patients was small, and the regression of fibrosis was modest [300].

More recently, the medical community’s hope was pinned on a new drug, a combination of ARB and NEP inhibitors (ARNI). Valsartan/sacubitril did not betray these expectations, showing improved outcomes (measured as mortality and hospitalization for HF) in HFrEF patients when compared with the ACE-I enalapril [301] (PARADIGM HF (NCT01035255). Neprilysin inhibition results in increased levels of several vasoactive molecules, including natriuretic peptides that are characterized by anti-inflammatory and anti-fibrotic properties [302]. Subgroup analysis showed that treatment decreased levels of biomarkers associated with fibrosis, suggesting that ARNI might reduce fibrosis in both HFrEF [303] and HFpEF [304] patients. Disappointingly, despite being efficacious in HFrEF, RAAS inhibition failed to demonstrate efficacy in HFpEF patients [305,306,307,308]. Even the combination of Valsartan/sacubitril did not significantly reduce the composite endpoint of CV death and total hospitalizations for HF (HHF) among patients with HF and ejection fraction (EF) >45%. However, in the prespecified subgroup analysis, a trend for improvement in HFpEF patients was present in patients in the lower EF range and in women (PARAGON-HF, NCT01920711) [308]. Based on this finding, US Food and Drug Administration (FDA) has approved the label expansion for sacubitril/valsartan “in patients with LVEF below normal”, specifying that “clinical judgment should be used in deciding whom to treat” [309].

The increased activation of the RAAS system is present in atherosclerosis as well as mentioned previously in this review. Several clinical trials demonstrate that RAAS inhibition is beneficial in patients with CAD. Specifically, ACE-I is associated with a decreased risk of MI in high-risk patients both in the EUROPE and HOPE trials [310,311]. This beneficial effect could only in part be attributed to the blood pressure-lowering effect since many of the patients enrolled in HOPE did not have hypertension, and the reduction of blood pressure with treatment was low. The athero-protective effect of RAAS inhibition may be due to its pleiotropic vascular effects, which include decreased Ang II-mediated oxidative stress, vasoconstriction, inflammation, endothelial dysfunction and VSMCs proliferation [311].

## 6. Conclusions and Future Perspectives

There is currently no drug directly targeting fibrosis approved in patients with cardiovascular disease. Several obstacles have limited drug development: Lack of ‘druggable’ targets, lack of efficacy, the lack of reliable biomarkers to detect fibrosis at the earliest stages, and finally, safety concerns.

As we demonstrate in this review, fibrosis could be beneficial or detrimental, depending on the context. After cardiac injury, like MI, fibrosis is critical in stabilizing the ventricle wall structure, given the quite limited regenerative potential of the adult myocardium [312]. However, in more chronic conditions, both in the context of HFrEF and HFpEF, fibrosis is detrimental to cardiac function and can increase the risk of arrhythmia [313]. HFrEF patients now have an arsenal of proven, effective therapies that promote clinical stability, reducing mortality and HF hospitalization [314]. On the contrary, HFpEF management is largely based on the treatment of risk factors and co-morbidities. Recently, the FDA approved empagliflozin, a SGLT2 inhibitor, as a treatment for both HFrEF and HFpEF. However, the effect of this drug is attenuated in patients with EF > 60% s [315,316], and no drugs to date have reduced CV or all-cause mortality in chronic heart failure at the higher EF range [317]. In this context, an anti-fibrotic therapy that reduces myocardial stiffness would be extremely attractive. However, considering the extensive overlap of pro-fibrotic signaling pathways in CFs and VSMCs, anti-fibrotic targeting could potentially pose a danger to HFpEF patients who have coronary atherosclerotic plaques.

This review summarizes the main signaling pathways that regulate ECM production by CFs and VSMCs of the fibrotic heart and atherosclerotic plaque, respectively. Although we here have referred to ECM production as a general term, fibrotic remodeling is not just a question of the amount of ECM but also the quality and composition of ECM. Thus, future research and targeting strategies should aim to tease out the ECM composition and signaling components that distinguish and govern “bad” and “good” fibrosis at these two cardiac locations as a basis for the development of future anti-fibrotic drugs. Thus, the ideal drug will promote beneficial effects on both myocardial stiffness and atherosclerotic plaque stability. Furthermore, many of the current key insights are generated in murine models where the prevention of fibrosis is examined. However, patients will already have some degree of fibrosis or an existing fibrous cap. Thus, anti-fibrotic drug strategies focus on stopping the further progression of fibrosis, and suitable animal models to study fibrosis progression should be utilized to a greater extent for testing the therapeutic effect of potential drugs. Finally, it will be interesting to translate and expand these findings from pre-clinical models into the emerging larger human cohort data coupled with novel technologies such as spatial transcriptomics and scRNAseq. Enlighted by these new types of highly insightful data, it is conceivable that efficient and safe anti-fibrotic treatments will be available within the near future.

## Data Availability

Data is contained within the article.
